# Synergistic Activity of Thymol with Commercial Antibiotics against Critical and High WHO Priority Pathogenic Bacteria

**DOI:** 10.3390/plants12091868

**Published:** 2023-05-02

**Authors:** Cristina Gan, Elisa Langa, Antonio Valenzuela, Diego Ballestero, M. Rosa Pino-Otín

**Affiliations:** Faculty of Health Sciences, Universidad San Jorge, 50830 Villanueva de Gállego, Zaragoza, Spain; cgan@usj.es (C.G.); elanga@usj.es (E.L.);

**Keywords:** thymol, antibiotics, synergy, *Staphylococcus aureus*, *Streptococcus agalactiae*, *Acinetobacter baumannii*, natural product

## Abstract

The use of synergistic combinations between natural compounds and commercial antibiotics may be a good strategy to fight against microbial resistance, with fewer side effects on human, animal and environmental, health. The antimicrobial capacity of four compounds of plant origin (thymol and gallic, salicylic and gentisic acids) was analysed against 14 pathogenic bacteria. Thymol showed the best antimicrobial activity, with MICs ranging from 125 µg/mL (for *Acinetobacter baumannii*, *Pasteurella aerogenes,* and *Salmonella typhimurium*) to 250 µg/mL (for *Bacillus subtilis*, *Klebsiella aerogenes*, *Klebsiella pneumoniae*, *Serratia marcescens*, *Staphylococcus aureus,* and *Streptococcus agalactiae)*. Combinations of thymol with eight widely used antibiotics were studied to identify combinations with synergistic effects. Thymol showed synergistic activity with chloramphenicol against *A. baumannii* (critical priority by the WHO), with streptomycin and gentamicin against *Staphylococcus aureus* (high priority by the WHO), and with streptomycin against *Streptococcus agalactiae*, decreasing the MICs of these antibiotics by 75% to 87.5%. The kinetics of these synergies indicated that thymol alone at the synergy concentration had almost no effect on the maximum achievable population density and very little effect on the growth rate. However, in combination with antibiotics at the same concentration, it completely inhibited growth, confirming its role in facilitating the action of the antibiotic. The time–kill curves indicated that all the combinations with synergistic effects were mainly bactericidal.

## 1. Introduction

The discovery of antibiotics (ABXs) was a true revolution for public health, and has saved millions of lives. However, their excessive consumption and irrational use have led to their dispersion in the environment and the emergence of ABX-resistant bacteria [1]. The World Health Organization (WHO) has declared that the emergence of multidrug-resistant (MDR) pathogens is one of the greatest threats to global health, food security, and development [2]. In recent decades, the consumption of ABXs has continued to grow. Between 2000 and 2010, ABX drug consumption increased by 36% (from 54,083,964,813 standard units in 2000 to 73,620,748,816 standard units in 2010) worldwide, with aminoglycosides as one of the most used [3]. This enormous quantity of ABXs, once consumed, passes into the wastewater where, in the best of cases, it reaches sewage treatment plants that do not eliminate these residues [4], and discharge them into watercourses. Levels in the ng/L range have been detected in effluents containing high concentrations of most ABXs that we study here, e.g., chloramphenicol (CHL) [5]. Some of these wastes become part of the sludge from wastewater treatment plants and end up being applied to soils as fertilizers [6]. ABXs have been detected in soils at different concentrations ranging from ng/kg to mg/kg [7]. For example, a concentration of 5.6 ng/kg of streptomycin (STM) was reported in US sandy loam soil after the addition of manure [8]. All this leads to a large dissemination of ABX residues in the environment, which will facilitate the selective pressure and the spread of resistance genes [9]. Resistance can arise from mutations that alter the bacterial molecular targets of the ABX. The difficulty in treating infections due to MDR pathogens makes it urgent to search for new antimicrobial substances with different mechanisms of action capable of producing less resistance and, if possible, with fewer side effects on human, animal, and environmental health, in line with the ‘‘One Health’’ strategy. The WHO has also developed an action plan to combat MDR strains, and one of the key points is the development of new antimicrobial products [10]. Therefore, many studies have focused on prospecting natural products to find new potential antimicrobial agents [11].

Many natural products from plants, especially essential oils (EOs), have been explored for the treatment and prevention of MDR bacteria [12,13]. Unfortunately, natural products usually have weaker antibiotic activity than common ABXs; therefore, it is difficult for them to effectively replace current ABXs in clinical practice. However, some plant-derived antimicrobial compounds have been shown to synergistically enhance antibiotic activity [14]. The synergistic interaction of natural compounds with already available ABXs may allow for the combination to be as effective as the ABX alone, and while maintaining the use of commercial ABXs, it lowers the minimum inhibitory concentration (MIC) of both the ABX and the natural product [15].

The use of lower concentrations of both agents offers important opportunities in the search for alternatives to the treatment of infectious diseases, as combinations with synergistic effects may reduce the probability of the emergence of bacterial resistance [16] while having effective pharmacological results [17]. Moreover, it may involve a reduction in ABX toxicity [18] with fewer side effects compared to those derived from high doses of synthetic drugs [19].

Thymol (2-isopropyl-5-methylphenol, THY) is one of the main phenolic monoterpenes found in EOs extracted from plants belonging to the Lamiaceae family, such as those of the genera *Thymus*, *Ocimum*, *Origanum*, *Satureja*, *Thymbra,* and *Monarda* [20,21,22,23]. It has a molecular weight of 150.22 g/mol and a solubility of 900 mg/L [24] and logP (o/w) = 3.3 [25], which indicates that it is a slightly water-soluble compound. Moreover, its pKa = 10.6 [26], which indicates that it is a molecule that at physiological pH 7.4 will be non-ionized. Essential oils of these plants have demonstrated antimicrobial properties primarily attributed to their main components, THY [20,21] among them. THY exhibits broad bioactivity [27]; especially, its antimicrobial activity has been quantitatively assessed on *Escherichia coli*, *Staphylococcus aureus*, *Listeria monocytogenes,* and *Bacillus subtilis* [28,29,30,31]. However, the antimicrobial effect of THY in combination with commercial ABXs has been much less explored. Other authors [14] have studied the interaction of THY with ampicillin, bacitracin, erythromycin, and penicillin in four ABX-resistant bacteria, finding synergistic effects in several cases, as with *Salmonella typhimurium* combined with ampicillin, tetracycline, penicillin, or erythromycin. Other authors have found synergistic activity between THY and other ABXs, such as vancomycin against *E. coli* [31], and antibiofilm activity in combinations of THY with three aminoglycosides against *Klebsiella pneumoniae* [32]. These studies indicate that THY presents favourable characteristics to be used in combination with ABXs in the treatment of infectious diseases, but their interaction with most of the ABXs used, as well as the synergistic effects on the numerous pathogenic bacteria of major clinical interest, have not yet been studied. The European Commission considers THY a low-risk product in consumption, and it is tested for use as a food flavouring. The Food and Drug Administration (FDA) has further classified THY as “generally safe’’ [27].

Another interesting group of plant secondary metabolites are the hydroxybenzoic acids, which are phenolic compounds characterized by an aromatic ring with an acid group and one or more hydroxyl groups. Among the representatives of this chemical family are salicylic acid (2-hydroxybenzoic acid, SA), gentisic acid (2,5-dihydroxybenzoic acid, GEA), and gallic acid (3,4,5-trihydroxybenzoic acid, GA). SA is a natural product that is frequently used in cosmetics because of its ability to promote exfoliation and of its anti-inflammatory and topical antibacterial activity [33]. Antibacterial activity against various bacterial strains, such as *E. coli* and *S. aureus* [34], has also been demonstrated. Similar to SA, GEA also exhibits antimicrobial activity against both Gram-positive and negative bacteria [35], and has antiarrhythmic, antirheumatic, analgesic, and anti-inflammatory properties [33]. GA has antioxidant, antimelanogenic [36], and antimicrobial properties, with demonstrated activity against *Enterococcus faecalis*, *S. aureus*, *E. coli,* and *Pseudomonas aeruginosa* among others [37].

The aim of this study is to explore combinations of natural products of plant origin with commercial ABXs in search of the ones with synergistic effects and with lower required doses of the ABX. For this purpose: (1) the MIC of four natural products and eight widely consumed ABXs are studied against 14 microbial strains responsible for numerous human and veterinary diseases and food spoilage; (2) from the natural products with the lowest MIC, combinations with ABXs are studied to identify synergistic combinations. For this purpose, bactericidal and bacteriostatic synergistic effects are quantified and the growth kinetics and time–kill curves of bacteria exposed to the most promising natural product/ABX combinations are analysed. Bacterial types were selected based on their clinical interest, as they cause some of the most common infections today [38,39], and on their potential severity and ability to generate resistance, according to the WHO’s list of priority pathogens [40].

## 2. Results

### 2.1. Antimicrobial Properties of Natural Products

The antibacterial activity of THY, GA, SA, and GEA against 14 microorganisms is shown in Table 1. THY had strong antimicrobial effects (See Material and Methods for the qualitative evaluation of the antimicrobial activity of the natural products tested) against seven out of the nine Gram-negative bacteria tested and against three out of the five Gram-positive bacteria, at concentrations below or equal to 500 µg/mL. The lowest MICs were 125 µg/mL for the Gram-negative *Acinetobacter baumannii*, *Pasteurella* aerogenes, and *S. typhimurium*. The values of the ratio between the minimum bactericidal concentration (MBC) and the MIC of THY showed that the activity was bactericidal in all cases (MBC/MIC ≤ 4) [41,42,43]. THY had higher MBC/MIC ratios for the Gram-positive cocci.

SA was the second most bioactive natural product, showing weak antibacterial activity against 12 out of the 14 bacteria tested, with MIC values between 1000 and 5000 µg/mL. *P. aerogenes* was the most sensitive strain to this compound (MIC = 625 µg/mL); the same strain was also the most sensitive with THY and GA. GEA and GA exhibited low to no antibacterial activity, with MIC values ranging between 1250 and 5000 µg/mL, and between 2500 and 5000 µg/mL, respectively. According to the MBC/MIC index, all three acids exhibited bactericidal activity.

The MICs of the ABXs are given in Table 2. These concentrations will be used to calculate the fractional inhibitory concentration index (FIC_I_) in the combinations with synergistic effects.

### 2.2. Synergies between Thymol and Antibiotics

**Figure 1:**
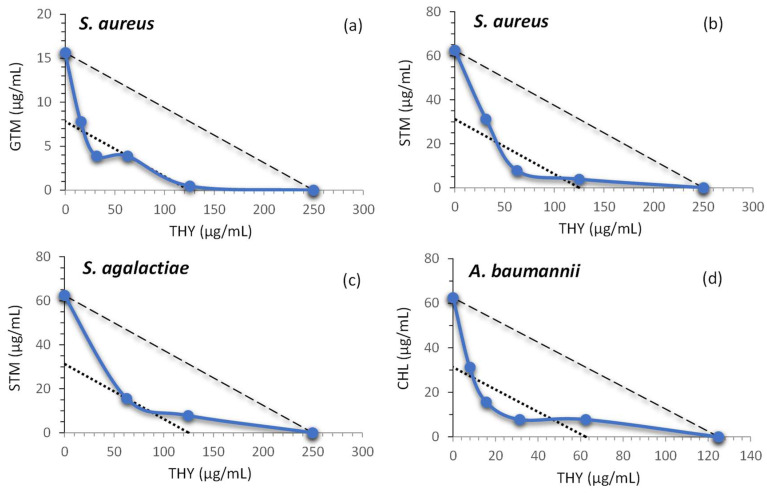
Isobolograms (blue solid line) of THY interactions with (**a**) GTM; (**b**) STM; (**c**) STM; (**d**) CHL that include synergistic effects. The THY concentration is represented on the x-axis and the different ABX concentrations on the y-axis. The MIC values are located on the respective axes (points where the isobologram intersects the coordinate axes). The straight “addition line” (upper dashed line), allows for the distinction of additive effects (above the straight line or in its immediate vicinity) from synergistic effects (concave isoboles below the line). It also has a line representing the synergy edge (lower dotted line). The points above or below the latter line represent synergistic combinations.

The FIC_I_s of the combinations of THY with the ABXs from the checkerboard test are shown in Table 3. The corresponding isobolograms of the combinations that showed one or more interactions with a FIC_I_ ≤ 0.5 are shown in Figure 1. Among the 30 combinations of THY with the ABXs, four showed synergism (FIC_I_ ≤ 0.5), 14 showed additivity (0.5 < FIC_I_ ≤ 1), and 12 showed no interaction (1 < FIC_I_ < 2). None of the combinations showed antagonistic effects (FIC_I_ ≥ 2).

Two of the most pronounced results were obtained with the combination of THY and STM against *S. aureus*, and THY and CHL against A. baumannii, both showing a significant synergistic effect (FIC_I_ = 0.375) and achieving an ABX dose reduction from 62.5 to 7.8 µg/mL (ABX dose reduction of 87.5%). Two other very promising results, with a four-fold dose reduction of ABX (75% dose reduction), were observed with THY and gentamycin (GTM) against *S. aureus* (FIC_I_ = 0.375), and THY and STM against *S. agalactiae* (FIC_I_ = 0.5). For all the other combinations tested, there were either additive effects or no interaction of the compounds (Table 3).

As shown in Figure 1a, the synergy of THY and GTM against *S. aureus* presented two points of synergistic interaction, with FIC_I_ values of 0.375 and 0.5 (in both cases, the ABX concentration was reduced to 3.9 µg/mL). The combination of THY with STM (Figure 1b,c) showed only one point of synergistic interaction when tested against both *S. aureus* and *S. agalactiae* (points above or below the lower dotted line). The reduction of STM concentration was greater in the case of *S. aureus*. Figure 1d shows how the combination of THY and CHL produced two interaction points with FIC_I_ = 0.375, one with a reduction of CHL to 7.8 μg/mL and the other to 15.6 μg/mL. In the cases where two combinations had the same FIC_I_, the one with the highest ABX reduction in its MIC was chosen for the kinetic tests.

### 2.3. Synergy Kinetics Study and Time–Kill Curves

**Figure 2:**
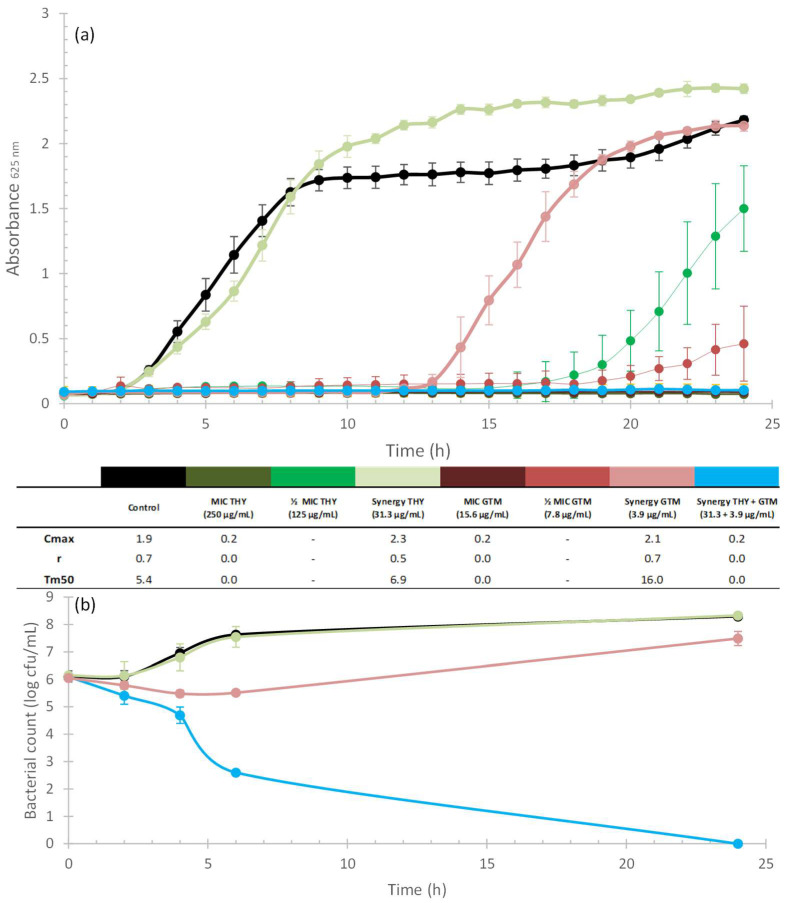
Kinetic assays and Cmax, r, and Tm50 values of THY (greenish curves) and GTM (reddish curves) alone and in combination (blue curves) against *S. aureus*; the darker the colour of the curve, the higher the concentration applied for the two compounds when tested alone. Black curves correspond to the control. (**a**) Growth kinetics assay. -: values achieved outside the studied range. Error bars are standard deviations (n = 4). (**b**) Time–kill curves. Error bars are standard deviations (n = 3).

**Figure 3:**
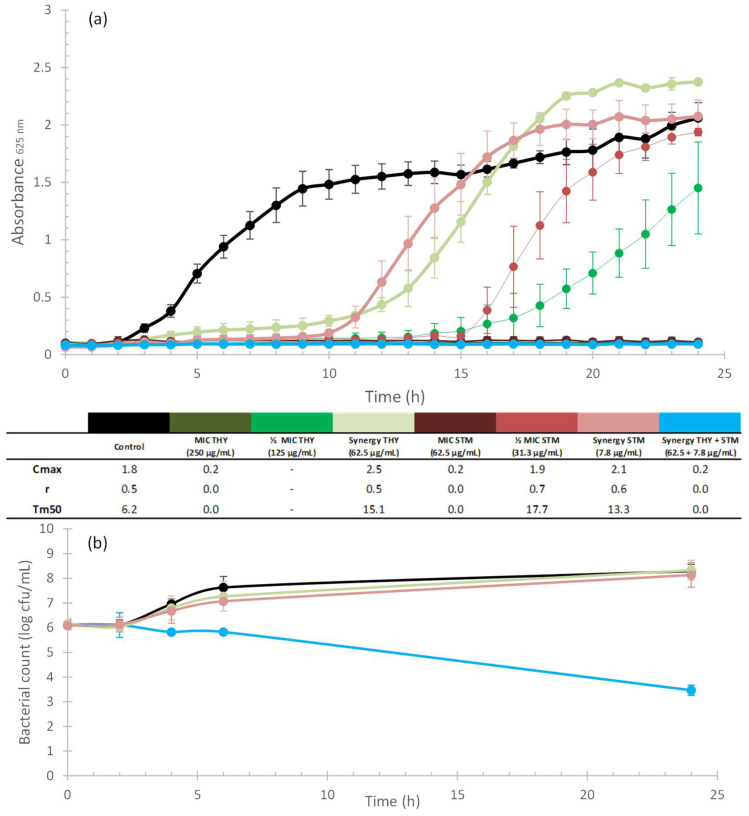
Kinetic assays and Cmax, r, and Tm50 values of THY (greenish curves) and STM (reddish curves) alone and in combination (blue curves) against *S. aureus*; the darker the colour of the curve, the higher the concentration applied for the two compounds when tested alone. Black curves correspond to the control. (**a**) Growth kinetics assay. -: values achieved outside the studied range. Error bars are standard deviations (n = 4). (**b**) Time–kill curves. Error bars are standard deviations (n = 3).

**Figure 4:**
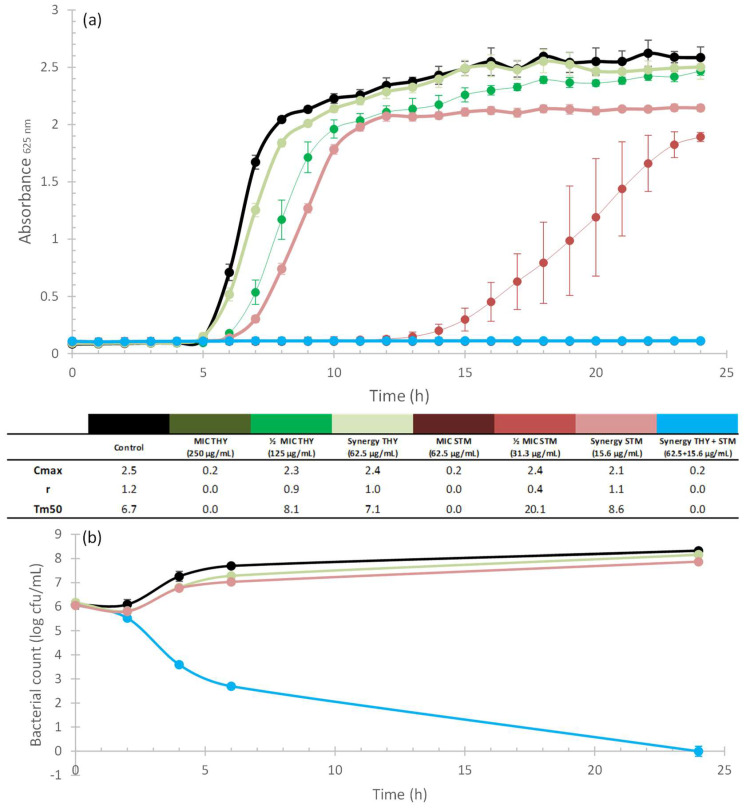
Kinetic assays and Cmax, r, and Tm50 values of THY (greenish curves) and STM (reddish curves) alone and in combination (blue curves) against *S. agalactiae*; the darker the colour of the curve, the higher the concentration applied for the two compounds when tested alone. Black curves correspond to the control. (**a**) Growth kinetics assay. Error bars are standard deviations (n = 4). (**b**) Time–kill curves. Error bars are standard deviations (n = 3).

**Figure 5:**
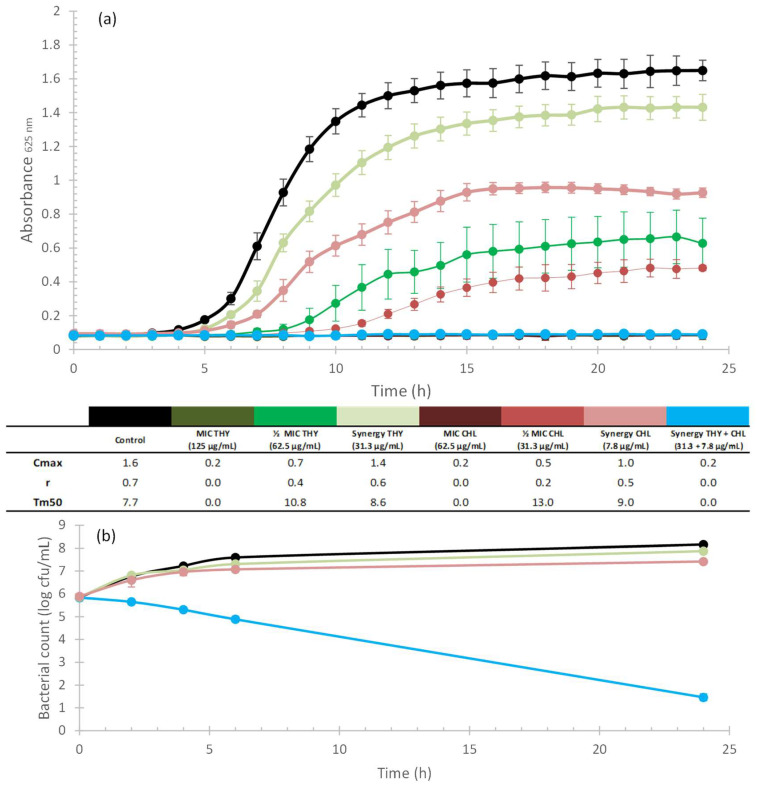
Kinetic assays and Cmax, r, and Tm50 values of THY (greenish curves) and CHL (reddish curves) alone and in combination (blue curves) against *A. baumannii*; the darker the colour of the curve, the higher the concentration applied for the two compounds when tested alone. Black curves correspond to the control. (**a**) Growth kinetics assay. Error bars are standard deviations (n = 4). (**b**) Time–kill curves. Error bars are standard deviations (n = 3).

Figure 2a, Figure 3a, Figure 4a and Figure 5a illustrate the growth kinetics of the synergistic combinations (blue line). The growth kinetics of ABXs alone (red lines) and THY alone (green lines), at different concentrations, are also shown. The curves have a greater colour intensity at higher concentrations (the darkest curve is the MIC concentration and the lightest one represents the synergistic concentration) for both ABX and THY. The control is represented by a black line. Cmax, r, and Tm50 values are included in a table below the graphs to better characterize the growth kinetics curves. Figure 2b, Figure 3b, Figure 4b and Figure 5b show the time–kill curves that present the mortality of the bacteria along the growth kinetics. As can be seen in all of the growth kinetics curves of the synergies (as well as those of the respective MICs of ABXs and THY), there was complete growth inhibition, so these curves are plotted horizontally on the *x*-axis.

The kinetic study of THY and GTM synergy against *S. aureus* is shown in Figure 2. Treatment with GTM at the synergistic concentration caused a decrease in the growth rate of *S. aureus* (Figure 2a), causing a delay in the exponential growth phase. Although exposure to THY alone at the synergistic concentration had little effect on the growth rate (r, Tm50) or on the maximum growth (Cmax) of the bacteria, it contributed to enhancing the effect of the ABX when combined, as the synergistic combination produced a total inhibition of growth over the 24 h studied. Figure 2b shows how at 6 h, the combination (blue line) had already killed a large part of the bacterial population, resulting in a reduction in the bacterial population of approximately 5 log_10_ CFU/mL compared to the control, and 2.9 log_10_ CFU/mL compared to GTM. This confirms the bactericidal effect of the combination and its synergistic effect. 

The kinetics of THY and STM against *S. aureus* (Figure 3a) indicates that both products at the synergistic concentration decrease the bacterial growth but they have little effect on bacterial Cmax values. At half the MIC, both products markedly slowed the growth rate of the bacteria, and neither product reached the stationary phase after 24 h. In Figure 3b, we can see that at 24 h, the combination produced a decrease in survivors of 8.29 log_10_ CFU/mL compared to the control, and 8.14 log_10_ CFU/mL compared to STM, thus demonstrating the bactericidal and synergistic effects, respectively.

Figure 4 shows the kinetics of THY and STM against *S. agalactiae*. The synergistic combination produced a total inhibition of growth throughout the 24 h studied (Figure 4a), whereas both products alone only slightly affected the Cmax (curves very similar to the control). If we look at the synergy curve (blue) in Figure 4b, it can be seen that the combination was able to kill bacteria very quickly (4 h), with a reduction in the bacterial population of 3.6 log_10_ CFU/mL compared to the control, and 3.17 log_10_ CFU/mL compared to STM.

The growth curves of *A. baumannii* are shown in Figure 5. The results show that both compounds at sub-MIC concentrations affected the Cmax of the bacteria in a concentration-dependent manner (Figure 5a). The time–kill curve of the synergy (Figure 5b) revealed a reduction in the bacterial population of 8.15 log_10_ CFU/mL in comparison to the control, and 7.42 log_10_ CFU/mL in comparison to CHL at 24 h.

## 3. Discussion

### 3.1. Antimicrobial Activity of the Tested Natural Products

THY had the highest antimicrobial activity of the four natural products chosen for this research, showing a strong antibacterial activity on 10 of the 13 tested bacteria, with MICs in the range of 125–250 µg/mL. It was particularly effective (125 µg/mL) against *A. baumannii*, *P. aerogenes,* and *S. typhimurium* (Table 1). These MIC ranges can be considered as “strong” activity according to the criteria for the qualitative estimation of the antimicrobial activity of natural products [44,45,46]. Although great variability in MIC data is reported in the literature due to differences in the strains, solvent concentrations, culture medium, and techniques used [47], published MIC values for some of the tested bacteria were quite similar to those obtained in this study. For example, in the case of *S. aureus* for THY, the reported MICs are 250 µg/mL [48] or very similar to our values, 156 µg/mL [49] and 310 µg/mL [50]. Furthermore, in the case of *E. coli*, other authors obtained similar MIC results to ours in response to THY, 400 µg/mL [28] or 250 µg/mL [48], or even identical (125 µg/mL) for *A. baumannii* [31]. To our knowledge, published MIC values for *Klebsiella* refer to *K. pneumonia*, and are similar to our value (250 µg/mL): up to 256 µg/mL against *K. pneumoniae* biofilms [51] or slightly higher (703 µg/mL) in other cases [52]. Previously reported MIC values of THY against four strains of *E. faecalis* are in the range of 1000 to 1200 µg/mL [37,53]. Hamoud et al. [31] tested *B. subtilis*, obtaining a slightly lower MIC (125 µg/mL), but higher values (420 µg/mL) have also been reported [54]. *L. monocytogenes* was found to be susceptible to THY, and reported MIC values are in the range of 125–800 µg/mL [28,30], which are lower than our value (MIC > 1000 µg/mL). To our knowledge, there are no MIC values reported for THY against *Serratia marcescens*, *P. aeruginosa*, *P. aerogenes,* or *S. agalactiae*; there are MIC values for THY-containing essential oils, but THY has a varying participation and coexists in them with other EO constituents that may contribute to the produced inhibitory effect; hence, such values are not comparable with those of our study. Finally, in the case of *Proteus mirabilis*, DMSO (solvent used) was toxic at the whole concentration range, making it impossible to test this bacterial strain with THY.

All three organic acids (GA, GEA, and SA) showed antimicrobial properties against most of the strains studied, but their biological activity was relatively low as most of the MICs were >800 µg/mL. These data agree with those reported by other authors. For example, Kalinowska et al. [35] obtained MIC values for GEA and GA that were very similar to our values (Table 1) for *E. coli*, *P. aeruginosa*, *S. aureus,* and *B. subtilis*. The only exception was *E. coli*, for which a MIC was not achieved with GA in this study. The results are also consistent with those of other authors [55] who tested both GA and GEA against *S. aureus*, *E. coli,* and *P. aeruginosa*. Neither showed any activity below 1000 µg/mL against *E. coli* or *P. aeruginosa*, in line with data presented herein. SA was found in a previous study to have the highest antimicrobial activity among 16 natural products, including flavonoids and organic acids against four bacteria [56]; MICs were considerably lower than those obtained in this study. The differences may be due to the fact that they used a 20% solution of DMSO to dilute the products, a concentration that we found in previous studies [57] to be toxic to most of the bacteria.

### 3.2. Behaviour of Thymol in Combination with Antibiotics

#### 3.2.1. Thymol Synergies with Antibiotics against Gram-Positive Cocci

The combinations between THY and the selected ABXs that were tested showed that this compound facilitates the action of the aminoglycosides GTM and STM against the Gram-positive cocci *S. aureus* and *S. agalactiae*. The combination of THY with these ABXs reduced the MIC by 75% in the case of GTM against *S. aureus* and of STM against *S. agalactiae*, and by 87.5% in the case of STM against *S. aureus*. Both aminoglycosides have the same mechanism of action and the same ribosomal target: they interfere with the initial steps of protein synthesis by altering the 30S portion of the prokaryotic ribosome, leading to the misreading of the mRNA triplets [58]. 

According to literature reports, the most likely mechanism of action of THY is its ability to alter cell membranes [59,60]. On the one hand, the hydrophilic part of the molecule interacts with the polar part of the bacterial cell membrane while the hydrophobic benzene ring and lipid side chains of THY interact with the hydrophobic part of phospholipids, causing a loss of membrane stability and alterations in its permeability [22,61,62]. Although this seems to be the main mechanism, other studies specify that it may also have internal targets and interact at the mitochondrial level, causing the disruption of adenosine triphosphate (ATP) synthesis and inducing the generation of Na^+^ and Ca^2+^ metabolic disturbances, leading to an excess of oxygen free radicals that cause cell death [63,64]. In *S. aureus* specifically, it has been described that THY is not only capable of disrupting the membrane and altering cellular homeostasis, but also affects the NADPH/NADP(+) balance in cells [61,65]. THY appears to be especially effective against Gram-positive bacteria, such as *S. aureus* and *S. agalactiae*. This group of bacteria has a thick peptidoglycan layer that lacks the outer membrane barrier of Gram-negative bacteria, and appears to be more permeable to small hydrophobic molecules [66,67]. However, the effects of THY on the two Gram-positive cocci were very different, although the MIC of THY against *S. agalactiae* and *S. aureus* was the same (250 µg/mL), THY had little effect on *S. agalactiae,* but decreased drastically the growth rate of *S. aureus* at 62.5 µg/mL, and even more at 125 µg/mL (r values dropped by up to 60%). These differences could indicate slightly different modes of action of the THY on the membrane of the two cocci or their internal targets. Since the composition of the bacterial cell membrane (on which THY appears mainly to act) is similar, it is likely that the different behaviour of THY against *S. aureus* and *S. agalactiae* is perhaps due to structural differences in the Gram-positive walls of these two bacteria and in the molecules covalently attached to the peptidoglycan. More specifically, *S. aureus* produces wall teichoic acids made of linear chains of ribitol phosphate [68], but there are no reports on the presence of similar types of poly (alditol phosphate) wall teichoic acids in the cell wall of streptococci, including *S. agalactiae* [69,70]. *S. agalactiae* has, in addition, two specific polysaccharides: the capsular polysaccharide (CPS) and the group B carbohydrate (GBC) [71,72].

The efficacy of THY, when combined with the two ABXs at synergistic concentrations, was maximal, completely inhibiting bacterial growth. This may indicate that the ability of THY to disrupt bacterial coatings probably facilitates the access of an ABX to its ribosomal target, making it much more effective. Membrane permeabilization by other plant-derived compounds, leading to increased absorption of ABXs, is a mechanism of action previously proposed for combinations with synergistic effects [73]. 

In the literature, there are reports of synergies of THY with ABXs other than the ones that we tested. For example, THY presents synergies with mupirocin against *S. aureus* in biofilms [74], and synergies of THY in combination with ABXs have also been described against *L. monocytogenes* [75].

#### 3.2.2. Thymol Synergies with Antibiotics against *Acinetobacter baumannii*

According to our results, THY at 125 µg/mL was able to completely inhibit the growth of *A. baumannii*. At the synergistic concentration (31.3 µg/mL), it was able to decrease only slightly both the bacterial growth rate and Cmax, following a behaviour similar to that of CHL when applied alone at the synergistic concentration (7.8 µg/mL). However, the ABX was notably more effective. When applied together at these sub-MIC concentrations, they were able to completely inhibit the growth of *A. baumannii* in a synergistic manner, resulting in THY being able to decrease the MIC of CHL by 87.5%. The THY + CHL combination, at synergistic concentrations, was bactericidal.

Many products of natural origin have been reported to possess considerable biocidal and/or biostatic activity against Gram-positive bacteria, but not so much against Gram-negative bacteria [76], mainly because Gram-negative bacteria have an outer membrane surrounding the cell wall peptidoglycan that is rich in lipopolysaccharides on its outer face, which may limit the diffusion of hydrophobic compounds [77]. In addition, MDR pumps, capable of extruding amphipathic molecules through the Gram-negative outer membrane, have been described [78]. However, according to our results, THY is probably able to cross the different coatings of this type of bacteria (outer membrane, cell wall, and cell membrane), enhancing the action of CHL. Helander et al. [79] described that THY can disintegrate the outer membrane of bacteria, so it can pass through this lining without problems until it reaches the cell membrane. In addition, the presence of outer membrane porins allows the passage of small hydrophobic molecules, such as THY. Interestingly, porins are downregulated in ABX-resistant strains [80]. On the other hand, the mechanism of action of CHL affects protein synthesis. It is capable of binding to the 50S subunit of bacterial ribosomes, inhibiting peptide bond formation, and thus preventing the elongation of the peptide chain under synthesis [58]. THY contributes to cell damage through its action on oxidative stress and other effects on cellular metabolism. For example, the citrate pathway and enzymes associated with ATP synthesis are inhibited in the Gram-negative bacterium *S. typhimurium* on exposure to THY [81]. Some studies indicate synergies of THY with other ABXs in other Gram-negative bacteria [51,75], but to our knowledge, no synergies of THY with CHL against *A. baumannii* have been previously described.

### 3.3. Relevance of Thymol Synergies with Gentamicin, Streptomycin, and Chloramphenicol

Both *S. aureus* and *A. baumannii* and, to a lesser extent, *S. agalactiae* are potent pathogens responsible for very serious diseases, many of them nosocomial and with an enormous capacity to disseminate resistance genes.

*A. baumannii* is one of the leading causes of hospital-acquired infections, especially in immunocompromised patients. Many of the strains isolated are resistant to all clinically available ABXs [82,83]. Multidrug resistance, combined with environmental resistance, makes *A. baumannii* strains potent nosocomial pathogens [84]. Given the above, *A. baumannii* has been declared by the WHO as a critical priority pathogen [40]. Therefore, new strategies to treat and manage infections caused by MDR *Acinetobacter* strains are urgently needed [85].

*S. aureus* can become an opportunistic pathogen, as it is one of the main causes of hospital-acquired infections, and it can cause significant morbidity and mortality, as well as high healthcare costs. Today, methicillin-resistant *S. aureus* (MRSA) is one of the most common bacteria responsible for outbreaks and hospital-acquired infections [86]. Due to these reasons, the WHO considers MRSA as a high-priority bacterium for which the development of new ABXs is needed [40].

*S. agalactiae* is the main pathogen of bovine mastitis, but it is also a human pathogen, especially for immunocompromised patients [87,88,89]. Although the acquisition of ABX resistance in streptococci has not been as critical as that detected in the other two bacteria, probably due to the much more limited horizontal spread of resistance genes [90], a multitude of cases occur worldwide [91,92]. 

Global ABX drug consumption is probably one of the main causes of ABX resistance. For example, a positive correlation between carbapenem consumption and aminoglycoside cross-resistance rates in *A. baumannii* [93] and in *S. aureus* [94] have been described. The large dissemination of ABX residues in the environment and its effects, including the spread of resistance genes, could be combated by using combinations of antimicrobials targeting different sites [95]. This is why the synergy of THY (which acts mainly on bacterial envelope membranes) with these three commercial ABXs (which target bacterial ribosomes) is so relevant, as it would reduce the occurrence of resistance by diversifying the target of action of the combined antimicrobials, as well as reducing the consumption of commercial ABXs. The reduction of commercial ABXs can also help minimize the impact of ABXs on the environment or on non-target organisms, including GTM, STM, and especially CHL, which is highly toxic to soil bacteria [96]. Broad-spectrum ABXs, such as GTM or CHL, can also be detrimental to human health as they affect the normal microbiota, causing dysbiosis [97]. In addition, ABXs targeting protein synthesis, such as GTM, STM, and CHL, can severely damage mitochondria and affect the normal physiological functions of cells [98,99]. Furthermore, in 2002, the FAO urged countries to stop using CHL in animal production (FAO, 2002). The Joint FAO/WHO Expert Committee on Food Additives (JECFA) concluded that the compound is genotoxic, meaning that it can cause genetic damage and possibly lead to cancer. Therefore, any development that minimizes the consumption of these ABXs can lead to improvements in the broader sense of One Health: human, animal, and environmental health.

### 3.4. Future Challenges

The challenge of the clinical (human and veterinary) application of synergistic combinations of commercial ABXs and THY requires the development of safe medical and health-related products that act effectively as resistance-modifying agents (RMAs) and that have a lower impact on health and on the environment than commercial ABXs. Although the safety and stability of the combination to be applied must be tested to avoid adverse THY–drug interactions, all three individual ABXs that were used have already been tested for safety and effectiveness for human use, and are widely marketed. Moreover, the EPA states that THY is considered a safe product for use in food for humans and does not provide any tolerance requirement for its residues in or on all food products [100]. THY is also listed as a food additive by the FDA [101]. In addition, the EPA states that the use of THY should be safe for terrestrial and aquatic non-target organisms [100].

Repeated exposure to EOs does not seem to show effects on bacterial sensitivity [102,103]. However, little information is available on the ability of pure natural products to generate resistance. Some studies on THY gave inconclusive results, although they suggest that it may generate some bacterial tolerance to *E. coli*, but not resistance, given the limited degree of increase in MICs in the mutants [104]. To our knowledge, no information on the resistance-generating capacity of synergies between pure natural products and antibiotics has been reported. Moreover, there are many studies analysing the environmental impact of ABXs on non-target organisms in water and soil [96,105,106], but fewer on the impacts of natural products [107,108]; hardly anything is known about the impact of synergies.

Although THY-based ABXs are not commercially available, the antimicrobial activity of THY against common oral pathogens [62,109] has led to its incorporation in formulations of some medical products. This is the case, for example, for Listerine^®^, one of the most popular mouthwashes worldwide [110], but also for Cervitec^®^ Plus or Hexidine^®^. These commercial products have been shown to have great benefits for oral health and do not generate side effects when properly used [111]. Whether any of the synergies we have described can contribute to increase the efficacy of similar preparations could be explored.

Another aspect to consider is the most appropriate application route. Since there may be mechanisms that can affect the activity of the synergy in vivo, for example the presence of serum proteins or mucus [73], a topical application of synergistic combinations is perhaps most easily applicable, reducing the ABX dose. There are already previous experiences of bandages, wound dressings, and hydrogels, among others, based on biopolymeric materials designed with THY as an additive [112,113,114]. THY nanoparticles with antimicrobial activity have also been developed, for example, against *S. aureus* [115].

The applications of THY in other fields, as in veterinary medicine [116,117] and aquaculture industry [118] have also been explored. The restriction of the prophylactic use of ABX growth promoters in animal production by EU regulation 2019/6 [119] focuses on minimizing the consumption of ABXs in this sector, one of the main generators of the spread of microbial resistance [120]. Synergies with phytocompounds that allow for a reduction of the dose of ABXs applied could be a key strategy in this regard. Finally, THY has been used as an antimicrobial in the food industry. One possible application of synergistic combinations would be as disinfectants to inhibit the formation of microbial biofilms on stainless steel surfaces [121].

## 4. Materials and Methods

### 4.1. Antimicrobial Compounds

Four natural products (see Figure S1 for the chemical structures of the tested natural products) known to have antimicrobial activity that were tested: thymol (THY), gallic acid (GA), gentisic acid (GEA), and salicylic acid (SA). The ABXs (a total of eight) tested were selected because they are some of the most widely used ABXs today representing different mechanisms of action. All of them were purchased from Acofarma (Barcelona, Spain) and Sigma- Aldrich (Darmstadt, Germany). Table 4 summarizes the detailed information for each compound.

### 4.2. Microorganisms

A total of 14 reference bacterial strains, responsible for highly prevalent human and veterinary diseases and food spoilage, were selected for this study, including both Gram-negative (*Acinetobacter baumannii* ATCC 19606, *Escherichia coli* ATCC 25922, *Klebsiella aerogenes* ATCC 13048, *Klebsiella pneumoniae* C6, *Pasteurella aerogenes* ATCC 27883, *Proteus mirabilis* ATCC 35659, *Pseudomonas aeruginosa* ATCC 27853, *Salmonella typhimurium* ATCC 13311, and *Serratia marcescens* ATCC 13880) and Gram-positive bacteria (*Bacillus subtilis* ATCC 6633, *Enterococcus faecalis* ATCC 19433, *Listeria monocytogenes* ATCC 7644, *Staphylococcus aureus* ATCC 9144, and *Streptococcus agalactiae* ATCC 12386). All microorganisms were purchased from Thermo Scientific (Dartford, United Kingdom) as freeze-dried Culti-loops™ bacteria, rehydrated and stored at −80 °C in cryovials (Deltalab S.L. Barcelona, Spain) until use. Rehydration and cultivation conditions for antimicrobial activity assays were carried out in accordance with ATCC and Thermo Scientific product sheet instructions for each strain (see Table S1).

### 4.3. Determination of the Antimicrobial Activity: Minimum Inhibitory Concentration (MIC) and Minimum Bactericidal Concentration (MBC)

To study the antimicrobial properties of the natural products and the ABXs, MICs were determined using the broth microdilution method in 96-well round-bottom microplates (Deltalab S.L. Barcelona, Spain), according to the Clinical and Laboratory Standards Institute (CLSI, M07-A9 2018) and ISO 207776-1 (2019) guidelines. The entire process was performed under sterile conditions in a flow chamber (Model MSC Advantage 1.2). Antimicrobial stock solutions of natural products and ABXs were prepared in distilled water (SIEMENS Ultra Clear™), except for THY, which was dissolved in 5% DMSO (CAS: 67-68-5), from Fisher Bioreagents (Madrid, Spain), with a purity ≥ 99.7%. The maximum solvent concentration per well was 2.5%. This concentration was tested to ensure that it did not affect bacterial growth. It was found to be innocuous for all of them, except for *P. mirabilis*; hence, THY could not be tested on this bacterium. The wells were filled with 100 µL of the appropriate medium for each bacterium (see Table S1). Then, 100 µL of natural product or ABX stock solution was added to the first column of each microplate, and serial two-fold dilutions were applied from columns 1 to 10, resulting in a final volume of 100 µL. A positive control for bacterial growth and a negative control for sterility were included in each experiment, in columns 11 and 12, respectively. Finally, 10 µL of inoculum were added to each well. Bacterial cultures were previously adjusted to the McFarland standard (CLSI, 2018) to reach an initial bacterial concentration per well of approximately 2.5 × 10^5^ CFU/mL, using a BioTek™ Synergy H1 hybrid multimode microplate reader (625 nm). Microplates were incubated (Incuterm, Trade Raypa^®^, bacteriological culture incubator) for 24 h at the appropriate temperature for each bacterium (Table S1). The MIC was considered as the lowest concentration that inhibited visible microbial growth according to CLSI guideline M07-A9 (2018). In order to achieve a more accurate measurement of microbial growth, the absorbance of each well was also measured at 625 nm using a microplate reader. Natural product activity was classified as strong (<400 µg/mL), moderate (400–800 µg/mL), or weak (>800 µg/mL) [44]. In addition, for natural products, the MBC was also studied; this is defined as the lowest concentration at which all bacteria are killed. For its determination, a 10 µL aliquot was taken from each non-growth column of the incubated 96-well plates and inoculated onto an agar plate. The plates were subsequently cultured for 24 h at the optimal growth temperature for each bacterial strain (Table S1) and monitored for any growth. The MBC/MIC ratio determines the bactericidal or bacteriostatic effect of the product on a bacterium. Antimicrobial substances are considered to have bactericidal activity when MBC/MIC ≤ 4 [41,42,43]; therefore, in this study the same criterion was followed for THY.

### 4.4. Determination of the Product Combination Behaviour

#### 4.4.1. Checkerboard Assays and Fractional Inhibitory Concentration Index

Selection of the combinations to be examined (among all possible ones) was made according to the following criteria: (1) the natural product should have the strongest antimicrobial activity among the four tested; (2) the ABX should have a MIC > 10 µg/mL (this increases the importance of reducing its effective dose than if it was already low).

The checkerboard method was used to measure potential synergies [14,122,123] between THY (drug A) and the tested ABXs (drug B). For the microdilution checkerboard test, THY was serially diluted vertically from columns 1 to 7 of 96-well microtiter plates. The corresponding ABX was then serially diluted horizontally from rows A to G of the plate, both products starting with a stock dilution corresponding to four times the MIC obtained for that product against a specific bacterium.

Next, the plates were inoculated with bacterial suspension adjusted to the McFarland standard prepared as discussed in Section 4.3. The plates were incubated at the optimal temperature for each strain (Table S1) for 24 h and then the absorbance (625 nm) was measured to evaluate the bacterial growth in the same way as described in Section 4.3.

To test the type of interaction between the drug combinations, the FIC_I_ was calculated for each combination, as follows [123,124]:

(1) $${FIC}_{I}={FIC}_{A}+{FIC}_{B}=\frac{{MIC}_{A+B}}{{MIC}_{A}}+\frac{{MIC}_{B+A}}{{MIC}_{B}}$$

where *FIC_A_* is the MIC of drug A (natural product) in the presence of the commercial ABX (drug B) (*MIC_A+B_*) divided by the MIC of drug A alone (*MIC_A_*). *FIC_B_* is the MIC of drug B in the presence of drug A (*MIC_B+A_*) divided by the MIC of the drug B alone (*MIC_B_*). According to the European Committee on antimicrobial susceptibility testing guidance [125], a *FIC_I_* value ≤ 0.5 indicates synergy; between 0.5 and 1 indicates additivity, whereas from >1 to 2, there is ‘‘no interaction’’ between the agents; *FIC_I_* values ≥ 2 imply antagonistic effects [126,127].

#### 4.4.2. Isobolograms

An isobologram (Figure 1) is a representation of the interaction between two substances. Isobolograms have been used to display the results of the checkerboard tests [128]. Unlike the growth kinetics or time–kill curves, this representation allows for the study of the interaction of ABXs and the natural product at several tested concentrations. Only isobolograms that showed in the checkerboard test one or more interactions with a FIC_I_ ≤ 0.5 have been plotted.

#### 4.4.3. Growth Kinetics Tests

For a better interpretation of the bacteriostatic effects of synergistic combinations (those with a FIC_I_ ≤ 0.5), growth kinetics tests were carried out. Bacterial cultures were adjusted to the McFarland standard, as previously described (Section 4.3). They were then exposed to different concentrations (MIC and sublethal concentrations) of natural products, commercial ABX, and a combination of both (according to the results obtained in the checkerboard test) in a 96-well microplate. They were then incubated at the corresponding temperature for each bacterium and absorbance measurements were taken every hour for 24 h. The results were plotted as absorbance vs. time to obtain growth curves (Figure 2a, Figure 3a, Figure 4a and Figure 5a). All experiments were performed in quadruplicate. Kinetic curves were fitted to a logistic model (Equation (2)) for sigmoid microbial growth [129] with the Excel Solver add-in (Microsoft 365):

(2) $$Absorbance=\frac{Cmax}{1+e^{b-rt}}$$

where *Cmax* is the carrying capacity, meaning the maximum achievable population density, *r* is the intrinsic rate of the population increase, and *b* is a fitting parameter. *Cmax*, *r,* and Tm50 (time in which half of the carrying capacity is reached) were calculated to characterize the kinetics of the different curves (see Figure 2a, Figure 3a, Figure 4a and Figure 5a).

#### 4.4.4. Time–Kill Curves

To study the bactericidal properties of the combinations, time–kill curves were obtained according to Hu et al. [126] (Figure 2b, Figure 3b, Figure 4b and Figure 5b). To this end, bacterial cultures (adjusted to the McFarland standard, as previously described) were exposed to THY and ABXs (alone and in combination) to a final volume of 10 mL, at concentrations of the selected synergistic combinations. Control tubes without antimicrobial agents were also included. Bacterial cultures exposed to the different concentrations were incubated at 37 °C for 24 h. Samples (100 µL) were collected at 0, 2, 4, 6, and 24 h. Serial dilutions of each sample were then prepared from 10^−1^ to 10^−7^, and 10 µL of each dilution was seeded on agar plates in triplicate. Following overnight incubation at 37 °C, the colonies were counted. The results were plotted as log_10_ CFU (y-axis) vs. time (x-axis) to obtain the time–kill curves. A product was considered bactericidal when the decrease in the number of survivors was greater than 3 log_10_ CFU/mL-fold compared to the control. In addition, synergy was defined as a ≥2 log_10_ CFU/mL-fold decrease by the combination compared to the most active single agent [130].

## 5. Conclusions

In this study, the antimicrobial properties of THY and three other products of natural origin (GA, SA, and GEA) were assessed on 14 Gram-positive and Gram-negative pathogenic bacteria. THY proved active against 10 of them. THY also showed synergistic effects when combined with GTM, STM, and CHL, and the reaction of some of these against bacteria, were considered as critical (*A. baumannii*) and of high priority (*S. aureus*) by the WHO, reducing the MIC of these ABXs by 75% to 87.5%. The study of the growth kinetics together with the time–kill curves seems to indicate that the possible role of THY in the synergies is to facilitate the access of the ABX, probably by altering the bacterial envelope. 

The results presented in this work show that THY as a product to be explored as an RMA, which may allow for a reduction in the consumption of ABXs in clinical and veterinary settings. This could contribute to reducing their impact on the environment and the generation of resistance, in line with the One Health strategy.

The identification and characterization of these synergies is the first step in a series to be made towards a healthier life and a safer environment. Although the ABXs are already marketed and THY is considered by the EPA and FDA as a safe product, the mode of application that would be the safest and most effective for treating human and veterinarian infections of these three bacteria is still a challenge.

## Figures and Tables

**Table 1:** Sensitivity of the microorganisms to the natural products examined.

Microorganism	Thymol			Gallic Acid			Salicylic Acid			Gentisic Acid		
	MIC	MBC	MBC/MIC	MIC	MBC	MBC/MIC	MIC	MBC	MBC/MIC	MIC	MBC	MBC/MIC
*Acinetobacter baumannii* ATCC 19606	125	250	2	5000	5000	1	1250	1250	1	2500	2500	1
*Bacillus subtilis* ATCC 6633	250	500	2	5000	5000	1	1250	1250	1	5000	5000	1
*Enterococcus faecalis* ATCC 19433	>1000	>1000	-	>5000	>5000	-	1250	1250	1	2500	2500	1
*Escherichia coli* ATCC 25922	500	500	1	>5000	>5000	-	1250	>1250	-	5000	>5000	-
*Klebsiella aerogenes* ATCC 13048	250	250	1	5000	5000	1	1250	1250	1	5000	5000	1
*Klebsiella pneumoniae * C6	250	250	1	>5000	>5000	-	1250	1250	1	5000	5000	1
*Listeria monocytogenes* ATCC 7644	>1000	>1000	-	>5000	>5000	-	1250	1250	1	2500	2500	1
*Pasteurella aerogenes* ATCC 27883	125	125	1	5000	5000	1	625	>1250	-	1250	1250	1
*Proteus mirabilis* ATCC 35659	-	-	-	5000	5000	1	>1250	>1250	-	5000	5000	1
*Pseudomona aeruginosa* ATCC 27853	>1000	>1000	-	5000	>5000	-	>1250	>1250	-	5000	>5000	-
*Salmonella typhimurium* ATCC 13311	125	125	1	5000	5000	1	1250	1250	1	2500	2500	1
*Serratia marcescens* ATCC 13880	250	250	1	5000	5000	1	1250	1250	1	2500	2500	1
*Staphylococcus aureus* ATCC 9144	250	1000	4	>5000	>5000	-	1250	>1250	-	5000	>5000	-
*Streptococcus agalactiae* ATCC 12386	250	1000	4	2500	2500	1	1250	1250	1	1250	1250	1

Concentration is given in µg/mL; -: insufficient data or test not carried out due to incompatibility with solvents.

**Table 2:** MIC values (µg/mL) of the commercial antibiotics that were examined.

Microorganism	Amoxicillin	Ampicillin	Chloramphenicol	Erythromycin	Gentamycin	Penicillin G	Streptomycin	Tetracycline
*A. baumannii*	250	250	62.5	15.6	15.6	500	250	0.8
*B. subtilis*	0.3	0.3	1.9	0.5	7.8	1.3	15.6	1.6
*E. coli*	7.8	7.8	7.8	250	31.3	-	125	0.8
*K. aerogenes*	>500	>500	31.3	62.5	0.8	-	3.9	2
*K. pneumoniae*	250	125	7.8	62.5	3.1	-	7.8	0.5
*P. aerogenes*	>500	>500	7.8	>500	6.3	-	7.8	7.8
*S. agalactiae*	0.2	0.2	15.6	0.5	7.8	0.2	62.5	0.2
*S. aureus*	0.6	0.2	31.3	0.6	15.6	1.3	62.5	62.5
*S. marcescens*	125	125	125	250	6.3	-	0.5	125
*S. typhimurium*	3.9	3.9	15.6	31.3	0.8	-	31.3	0.5

-: not tested.

**Table 3:** FIC_I_ values of thymol—antibiotics combinations.

Microorganism	Commercial ABX	MIC THY in Combination	MIC ABX in Combination	FIC_I_ *	Interpretation
*A. baumannii*	AMO	62.5	125	1	Additivity
	AMP	62.5	62.5	0.75	Additivity
	CHL	31.3	7.8	0.375	Synergy
	ERY	125	15.6	2	No interaction
	GTM	62.5	1	0.56	Additivity
	PEN	125	500	2	No interaction
	STM	62.5	125	1	Additivity
*B. subtilis*	STM	125	7.8	1	Additivity
*E. coli*	ERY	250	7.8	0.53	Additivity
	GTM	250	1.9	0.56	Additivity
	STM	250	7.8	0.56	Additivity
*K. aerogenes*	CHL	15.6	15.6	0.56	Additivity
	ERY	250	62.5	2	No interaction
*K. pneumoniae*	AMO	250	250	2	No interaction
	AMP	250	125	2	No interaction
	ERY	250	62.5	2	No interaction
*S. agalactiae*	CHL	250	15.6	2	No interaction
	STM	62.5	15.6	0.5	Synergy
*S. aureus*	CHL	250	31.3	2	No interaction
	GTM	31.3	3.9	0.375	Synergy
	STM	62.5	7.8	0.375	Synergy
	TC	250	62.5	2	No interaction
*S. marcescens*	AMO	250	125	2	No interaction
	AMP	250	125	2	No interaction
	CHL	125	62.5	1	Additivity
	ERY	125	125	1	Additivity
	TC	250	125	2	No interaction
*S. typhimurium*	CHL	62.5	3.9	0.75	Additivity
	ERY	62.5	15.6	1	Additivity
	STM	62.5	15.6	1	Additivity

Concentration is given in µg/mL; * FIC_I_ values are calculated according Equation (1).

**Table 4:** Information on the antimicrobial compounds used for the antibacterial tests.

Antibiotic/Natural Product	Abbreviation	Chemical Family	CAS Number	Supplier	Purity	Molecular Weight (g/mol)
Gentamycin	GTM	Aminoglycosides	1403-66-3	Acofarma	≥97%	447.6
Streptomycin	STM		57-92-1		≥97%	581.6
Chloramphenicol	CHL	Amphenicols	56-75-7		97.5%	323.1
Amoxicillin	AMO	Beta-lactams	26787-78-0	Sigma- Aldrich	96–102%	365.4
Ampicillin	AMP		69-53-4		≥90%	394.4
Penicillin G	PEN		69-57-8		96–102%	356.4
Erythromycin	ERY	Macrolides	114-07-8	Acofarma	95.9%	733.9
Thymol	THY	Monoterpenes	89-83-8	Sigma- Aldrich	100%	150.2
Gallic acid	GA	Phenolic acids	149-91-7		100%	170.1
Gentisic acid	GEA		490-79-9		98%	154.1
Salicylic acid	SA		69-72-7		100%	138.1
Tetracycline chlorhydrate	TC	Tetracyclines	64-75-5	Acofarma	99.2%	444.4

## Data Availability

Data is contained within the article or supplementary material.

## Supplementary Materials

The following supporting information can be downloaded at: https://www.mdpi.com/article/10.3390/plants12091868/s1, Table S1: Microorganisms’ reference and culture conditions according to ATCC datasheets for each microorganism. Figure S1: Chemical structures of the tested natural products.

## References

[1] Markowicz A., Bondarczuk K., Cycon M., Sulowicz S. (2021). Land application of sewage sludge: Response of soil microbial communities and potential spread of antibiotic resistance. Environ. Pollut..

[2] WHO (2015). Antimicrobial Resistance: Global Report on Surveillance.

[3] Van Boeckel T.P., Gandra S., Ashok A., Caudron Q., Grenfell B.T., Levin S.A., Laxminarayan R. (2014). Global antibiotic consumption 2000 to 2010: An analysis of Cross Mark 742 national pharmaceutical sales data. Lancet Infect. Dis..

[4] Zhang T., Li B. (2011). Occurrence, transformation, and fate of antibiotics in municipal wastewater treatment plants. Crit. Rev. Environ. Sci. Technol..

[5] Ruan Y., Wu R., Lam J.C.W., Zhang K., Lam P.K.S. (2019). Seasonal occurrence and fate of chiral pharmaceuticals in different sewage treatment systems in Hong Kong: Mass balance, enantiomeric profiling, and risk assessment. Water Res..

[6] Du L., Liu W. (2012). Occurrence, fate, and ecotoxicity of antibiotics in agro-ecosystems. A review. Agron. Sustain. Dev..

[7] Santas-Miguel V., Arias-Estevez M., Diaz-Ravina M., Fernandez-Sanjurjo M.J., Alvarez-Rodriguez E., Nunez-Delgado A., Fernandez-Calvino D. (2020). Interactions between soil properties and tetracycline toxicity affecting to bacterial community growth in agricultural soil. Appl. Soil Ecol..

[8] Cycon M., Mrozik A., Piotrowska-Seget Z. (2019). Antibiotics in the soil environment-degradation and their impact on microbial activity and diversity. Front. Microbiol..

[9] Tripathi V., Cytryn E., Venter H. (2017). Impact of anthropogenic activities on the dissemination of antibiotic resistance across ecological boundaries. Essays Biochem..

[10] WHO (2015). Global Action Plan on Antimicrobial Resistance.

[11] Ali S.M., Siddiqui R., Khan N.A. (2018). Antimicrobial discovery from natural and unusual sources. J. Pharm. Pharmacol..

[12] Salam A.M., Quave C.L. (2018). Opportunities for plant natural products in infection control. Curr. Opin. Microbiol..

[13] Burt S. (2004). Essential oils: Their antibacterial properties and potential applications in foods—A review. Int. J. Food Microbiol..

[14] Palaniappan K., Holley R.A. (2010). Use of natural antimicrobials to increase antibiotic susceptibility of drug resistant bacteria. Int. J. Food Microbiol..

[15] Ayaz M., Ullah F., Sadiq A., Ullah F., Ovais M., Ahmed J., Devkota H.P. (2019). Synergistic interactions of phytochemicals with antimicrobial agents: Potential strategy to counteract drug resistance. Chem.-Biol. Interact..

[16] Lewis K., Ausubel F.M. (2006). Prospects for plant-derived antibacterials. Nat. Biotechnol..

[17] Cheesman M.J., Ilanko A., Blonk B., Cock I.E. (2017). Developing new antimicrobial therapies: Are synergistic combinations of plant extracts/compounds with conventional antibiotics the solution?. Pharmacogn. Rev..

[18] Aiyegoro O.A., Okoh A.I. (2009). Use of bioactive plant products in combination with standard antibiotics: Implications in antimicrobial chemotherapy. J. Med. Plants Res..

[19] Ayaz M., Sadiq A., Wadood A., Junaid M., Ullah F., Khan N.Z. (2019). Cytotoxicity and molecular docking studies on phytosterols isolated from *Polygonum hydropiper* L.. Steroids.

[20] Karapinar M., Aktug S.E. (1987). Inhibition of foodborne pathogens by thymol, eugenol, menthol and anethole. Int. J. Food Microbiol..

[21] Lee S.J., Umano K., Shibamoto T., Lee K.G. (2005). Identification of volatile components in basil (*Ocimum basilicum* L.) and thyme leaves (Thymus vulgaris L.) and their antioxidant properties. Food Chem..

[22] Tohidpour A., Sattari M., Omidbaigi R., Yadegar A., Nazemi J. (2010). Antibacterial effect of essential oils from two medicinal plants against Methicillin-resistant Staphylococcus aureus (MRSA). Phytomedicine.

[23] Vokou D., Kokkini S., Bessiere J.M. (1993). Geographic-variation of Greek oregano (*Origanum-vulgare* ssp. *hirtum*) essential oils. Biochem. Syst. Ecol..

[24] Yalkowsky S.H., He Y., Jain P. (2010). Handbook of Aqueous Solubility Data Second Edition.

[25] Hansch C., Leo A., Hoekman D. (1996). Exploring QSAR—Hydrophobic, electronic, and steric constants. J. Med. Chem..

[26] Serjeant E.P., Dempsey B. (1979). Ionisation Constants of Organic Acids in Aqueous Solution.

[27] Marchese A., Orhan I.E., Daglia M., Barbieri R., Di Lorenzo A., Nabavi S.F., Gortzi O., Izadi M., Nabavi S.M. (2016). Antibacterial and antifungal activities of thymol: A brief review of the literature. Food Chem..

[28] Cusimano M.G., Di Stefano V., La Giglia M., Lo Presti V.D.M., Schillaci D., Pomilio F., Vitale M. (2020). Control of growth and persistence of listeria monocytogenes and beta-lactam-resistant escherichia coli by thymol in food processing settings. Molecules.

[29] Heckler C., Sant’anna V., Brandelli A., Malheiros P.S. (2021). Combined effect of carvacrol, thymol and nisin against *Staphylococcusaureus* and Salmonella Enteritidis. An. Acad. Bras. Cienc..

[30] Chen H., Zhong Q. (2017). Lactobionic acid enhances the synergistic effect of nisin and thymol against Listeria monocytogenes Scott A in tryptic soy broth and milk. Int. J. Food Microbiol..

[31] Hamoud R., Zimmermann S., Reichling J., Wink M. (2014). Synergistic interactions in two-drug and three-drug combinations (thymol, EDTA and vancomycin) against multi drug resistant bacteria including *E. coli*. Phytomedicine.

[32] Aleksic Sabo V., Nikolic I., Mimica-Dukic N., Knezevic P. (2021). Anti-Acinetobacter baumanniiactivity of selected phytochemicals alone, in binary combinations and in combinations with conventional antibiotics. Nat. Prod. Res..

[33] Khadem S., Marles R.J. (2010). Monocyclic phenolic acids; hydroxy- and polyhydroxybenzoic acids: Occurrence and recent bioactivity studies. Molecules.

[34] Song X., Li R., Zhang Q., He S., Wang Y. (2022). Antibacterial effect and possible mechanism of salicylic acid microcapsules against Escherichia coli and Staphylococcus aureus. Int. J. Environ. Res. Public Health.

[35] Kalinowska M., Golebiewska E., Swiderski G., Meczynska-Wielgosz S., Lewandowska H., Pietryczuk A., Cudowski A., Astel A., Swislocka R., Samsonowicz M. (2021). Plant-derived and dietary hydroxybenzoic acids—A comprehensive study of structural, anti-/pro-oxidant, lipophilic, antimicrobial, and cytotoxic activity in MDA-MB-231 and MCF-7 Cell Lines. Nutrients.

[36] Kim Y.-J. (2007). Antimelanogenic and antioxidant properties of gallic acid. Biol. Pharm. Bull..

[37] Gutierrez-Fernandez J., Garcia-Armesto M.R., Alvarez-Alonso R., del Valle R., de Arriaga D., Rua J. (2013). Antimicrobial activity of binary combinations of natural and synthetic phenolic antioxidants against *Enterococcus faecalis*. J. Dairy Sci..

[38] Ghadiri H., Vaez H., Khosravi S., Soleymani E. (2012). The antibiotic resistance profiles of bacterial strains isolated from patients with hospital-acquired bloodstream and urinary tract infections. Crit. Care Res. Pract..

[39] Gonzalez-Torralba A., Garcia-Esteban C., Alos J.-I. (2018). Enteropathogens and antibiotics. Enferm. Infecc. Microbiol. Clin..

[40] WHO (2017). WHO Priority Pathogens List for R&D of New Antibiotics.

[41] Adrar N., Oukil N., Bedjou F. (2016). Antioxidant and antibacterial activities of *Thymus numidicus* and *Salvia officinalis* essential oils alone or in combination. Ind. Crops Prod..

[42] Kuate C.R.T., Ndezo B.B., Dzoyem J.P. (2021). Synergistic antibiofilm effect of thymol and piperine in combination with aminoglycosides antibiotics against four *Salmonella enterica* Serovars. Evid.-Based Complement. Altern. Med..

[43] Pankey G.A., Sabath L.D. (2004). Clinical relevance of bacteriostatic versus bactericidal mechanisms of action in the treatment of gram-positive bacterial infections. Clin. Infect. Dis..

[44] Taguri T., Tanaka T., Kouno I. (2006). Antibacterial spectrum of plant polyphenols and extracts depending upon hydroxyphenyl structure. Biol. Pharm. Bull..

[45] Rios J.L., Recio M.C. (2005). Medicinal plants and antimicrobial activity. J. Ethnopharmacol..

[46] Bubonja-Sonje M., Knezevic S., Abram M. (2020). Challenges to antimicrobial susceptibility testing of plant-derived polyphenolic compounds. Arh. Za Hig. Rada I Toksikol.-Arch. Ind. Hyg. Toxicol..

[47] Mauriello E., Ferrari G., Donsi F. (2021). Effect of formulation on properties, stability, carvacrol release and antimicrobial activity of carvacrol emulsions. Colloids Surf. B-Biointerfaces.

[48] Guarda A., Rubilar J.F., Miltz J., Jose Galotto M. (2011). The antimicrobial activity of microencapsulated thymol and carvacrol. Int. J. Food Microbiol..

[49] Wattanasatcha A., Rengpipat S., Wanichwecharungruang S. (2012). Thymol nanospheres as an effective anti-bacterial agent. Int. J. Pharm..

[50] Trombetta D., Castelli F., Sarpietro M.G., Venuti V., Cristani M., Daniele C., Saija A., Mazzanti G., Bisignano G. (2005). Mechanisms of antibacterial action of three monoterpenes. Antimicrob. Agents Chemother..

[51] Bisso Ndezo B., Tokam Kuate C.R., Dzoyem J.P. (2021). Synergistic antibiofilm efficacy of thymol and piperine in combination with three aminoglycoside antibiotics against *Klebsiella pneumoniae* biofilms. Can. J. Infect. Dis. Med. Microbiol..

[52] Abbaszadeh S., Sharifzadeh A., Shokri H., Khosravi A.R., Abbaszadeh A. (2014). Antifungal efficacy of thymol, carvacrol, eugenol and menthol as alternative agents to control the growth of food-relevant fungi. J. Mycol. Med..

[53] Mazurova J., Kukla R., Rozkot M., Lustykova A., Slehova E., Sleha R., Lipensky J., Opletal L. (2015). Use of natural substances for boar semen decontamination. Vet. Med..

[54] Falcone P., Speranza B., Del Nobile M.A., Corbo M.R., Sinigaglia M. (2005). Study on the antimicrobial activity of thymol intended as a natural preservative. J. Food Prot..

[55] Vandal J., Abou-Zaid M.M., Ferroni G., Leduc L.G. (2015). Antimicrobial activity of natural products from the flora of Northern Ontario, Canada. Pharm. Biol..

[56] Adamczak A., Ozarowski M., Karpinski T.M. (2020). Antibacterial activity of some flavonoids and organic acids widely distributed in plants. J. Clin. Med..

[57] Pino-Otin M.R., Gan C., Terrado E., Sanz M.A., Ballestero D., Langa E. (2022). Antibiotic properties of *Satureja montana* L. hydrolate in bacteria and fungus of clinical interest and its impact in non-target environmental microorganisms. Sci. Rep..

[58] Madigan T.M., Martinko J.M., Bender K.S., Buckley D.H., Stahl S.A. (2015). Brock. Biología de los Microorganismos.

[59] Chauhan A.K., Kang S.C. (2014). Thymol disrupts the membrane integrity of *Salmonella* ser. *typhimurium* in vitro and recovers infected macrophages from oxidative stress in an ex vivo model. Res. Microbiol..

[60] Rhayour K., Bouchikhi T., Tantaoui-Elaraki A., Sendide K., Remmal A. (2003). The mechanism of bactericidal action of oregano and clove essential oils and of their phenolic major components on *Escherichia coli* and *Bacillus subtilis*. J. Essent. Oil Res..

[61] Li Q., Huang K.X., Pan S., Su C., Bi J., Lu X. (2022). Thymol disrupts cell homeostasis and inhibits the growth of *Staphylococcus aureus*. Contrast Media Mol. Imaging.

[62] Kachur K., Suntres Z. (2020). The antibacterial properties of phenolic isomers, carvacrol and thymol. Crit. Rev. Food Sci. Nutr..

[63] Baldissera M.D., Souza C.F., De Matos A.F.I.M., Doleski P.H., Baldisserotto B., Da Silva A.S., Monteiro S.G. (2018). Blood-brain barrier breakdown, memory impairment and neurotoxicity caused in mice submitted to orally treatment with thymol. Environ. Toxicol. Pharmacol..

[64] Cheng Y.-W., Kong X.-W., Wang N., Wang T.-T., Chen J., Shi Z.Q. (2020). Thymol confers tolerance to salt stress by activating anti-oxidative defense and modulating Na+ homeostasis in rice root. Ecotoxicol. Environ. Saf..

[65] Zhou W., Wang Z., Mo H., Zhao Y., Li H., Zhang H., Hu L., Zhou X. (2019). Thymol mediates bactericidal activity against *Staphylococcus aureus* by targeting an aldo-keto reductase and consequent depletion of NADPH. J. Agric. Food Chem..

[66] Silhavy T.J., Kahne D., Walker S. (2010). The bacterial cell envelope. Cold Spring Harb. Perspect. Biol..

[67] Hyldgaard M., Mygind T., Meyer R.L. (2012). Essential oils in food preservation: Mode of action, synergies, and interactions with food matrix components. Front. Microbiol..

[68] Neuhaus F.C., Baddiley J. (2003). A continuum of anionic charge: Structures and functions of D-Alanyl-Teichoic acids in gram-positive bacteria. Microbiol. Mol. Biol. Rev..

[69] Deng L.Y., Kasper D.L., Krick T.P., Wessels M.R. (2000). Characterization of the linkage between the type III capsular polysaccharide and the bacterial cell wall of group B *Streptococcus*. J. Biol. Chem..

[70] Decueninck B.J., Shockman G.D., Swenson R.M. (1982). Group-B, Type-III Streptococcal cell-wall—Composition and structural aspects revealed through endo-n-acetylmuramidase-catalyzed hydrolysis. Infect. Immun..

[71] Beaussart A., Pechoux C., Trieu-Cuot P., Hols P., Mistou M.-Y., Dufrene Y.F. (2014). Molecular mapping of the cell wall polysaccharides of the human pathogen *Streptococcus agalactiae*. Nanoscale.

[72] Yother J. (2011). Capsules of *Streptococcus pneumoniae* and other bacteria: Paradigms for polysaccharide biosynthesis and regulation. Annu. Rev. Microbiol..

[73] Langeveld W.T., Veldhuizen E.J.A., Burt S.A. (2014). Synergy between essential oil components and antibiotics: A review. Crit. Rev. Microbiol..

[74] Kifer D., Muzinic V., Klaric M.S. (2016). Antimicrobial potency of single and combined mupirocin and monoterpenes, thymol, menthol and 1,8-cineole against *Staphylococcus aureus* planktonic and biofilm growth. J. Antibiot..

[75] Liu Q., Niu H., Zhang W., Mu H., Sun C., Duan J. (2015). Synergy among thymol, eugenol, berberine, cinnamaldehyde and streptomycin against planktonic and biofilm-associated food-borne pathogens. Lett. Appl. Microbiol..

[76] Aldulaimi O.A. (2017). General overview of phenolics from plant to laboratory, good antibacterials or not. Pharmacogn. Rev..

[77] Garvey M.I., Rahman M.M., Gibbons S., Piddock L.J.V. (2011). Medicinal plant extracts with efflux inhibitory activity against Gram-negative bacteria. Int. J. Antimicrob. Agents.

[78] Lewis K. (2001). In search of natural substrates and inhibitors of MDR pumps. J. Mol. Microbiol. Biotechnol..

[79] Helander I.M., von Wright A., MattilaSandholm T.M. (1997). Potential of lactic acid bacteria and novel antimicrobials against gram-negative bacteria. Trends Food Sci. Technol..

[80] Fernando D., Zhanel G., Kumar A. (2013). Antibiotic resistance and expression of resistance-nodulation-division pump- and outer membrane porin-encoding genes in Acinetobacter species isolated from Canadian hospitals. Can. J. Infect. Dis. Med. Microbiol..

[81] Di Pasqua R., Mamone G., Ferranti P., Ercolini D., Mauriello G. (2010). Changes in the proteome of *Salmonella enterica* serovar Thompson as stress adaptation to sublethal concentrations of thymol. Proteomics.

[82] Valencia R., Arroyo L.A., Conde M., Aldana J.M., Torres M.-J., Fernandez-Cuenca F., Garnacho-Montero J., Cisneros J.M., Ortiz C., Pachon J. (2009). Nosocomial outbreak of infection with pan-drug-resistant acinetobacter baumannii in a tertiary care university hospital. Infect. Control Hosp. Epidemiol..

[83] Van Looveren M., Goossens H., Grp A.S. (2004). Antimicrobial resistance of *Acinetobacter* spp. in Europe. Clin. Microbiol. Infect..

[84] Montagu A., Joly-Guillou M.-L., Rossines E., Cayon J., Kempf M., Saulnier P. (2016). Stress conditions induced by carvacrol and cinnamaldehyde on *Acinetobacter baumannii*. Front. Microbiol..

[85] Weber B.S., Harding C.M., Feldman M.F. (2016). Pathogenic Acinetobacter: From the cell surface to infinity and beyond. J. Bacteriol..

[86] Uhlemann A.-C., Otto M., Lowy F.D., Deleo F.R. (2014). Evolution of community- and healthcare-associated methicillin-resistant *Staphylococcus aureus*. Infect. Genet. Evol..

[87] Fry R.M. (1938). Fatal infections by haemolytic Streptococcus Group B. Lancet.

[88] Edwards M.S. (1990). Group-B Streptococcal infections. Pediatr. Infect. Dis. J..

[89] Edmond K.M., Kortsalioudaki C., Scott S., Schrag S.J., Zaidi A.K.M., Cousens S., Heath P.T. (2012). Group B streptococcal disease in infants aged younger than 3 months: Systematic review and meta-analysis. Lancet.

[90] Haenni M., Lupo A., Madec J.-Y. (2018). Antimicrobial resistance in *Streptococcus* spp.. Microbiol. Spectr..

[91] Barros R.R. (2021). Antimicrobial resistance among beta-hemolytic streptococcus in Brazil: An overview. Antibiotics.

[92] Khademi F., Sahebkar A. (2020). Group B streptococcus drug resistance in pregnant women in Iran: A meta-analysis. Taiwan. J. Obstet. Gynecol..

[93] Cao J., Song W., Gu B., Mei Y.-n., Tang J.-p., Meng L., Yang C.-q., Wang H., Zhou H. (2013). Correlation between carbapenem consumption and antimicrobial resistance rates of *Acinetobacter baumannii* in a University-affiliated hospital in China. J. Clin. Pharmacol..

[94] Westh H., Zinn C.S., Rosdahl V.T., Grp S.S. (2004). An international multicenter study of antimicrobial resistance and typing of hospital *Staphylococcus aureus* isolates from 15 hospitals in 14 countries. Microb. Drug Resist..

[95] Foster T.J. (2017). Antibiotic resistance in *Staphylococcus aureus*. Current status and future prospects. FEMS Microbiol. Rev..

[96] Pino-Otin M.R., Ferrando N., Ballestero D., Langa E., Roig F.J., Terrado E.M. (2022). Impact of eight widely consumed antibiotics on the growth and physiological profile of natural soil microbial communities. Chemosphere.

[97] Keeney K.M., Yurist-Doutsch S., Arrieta M.-C., Finlay B.B. (2014). Effects of antibiotics on human microbiota and subsequent disease. Annu. Rev. Microbiol..

[98] Rai A., Khairnar K. (2021). Overview of the risks of *Staphylococcus aureus* infections and their control by bacteriophages and bacteriophage-encoded products. Braz. J. Microbiol..

[99] Singh R., Sripada L., Singh R. (2014). Side effects of antibiotics during bacterial infection: Mitochondria, the main target in host cell. Mitochondrion.

[100] EPA Thymol; Exemption From the Requirement of a Tolerance. https://www.federalregister.gov/documents/2022/09/07/2022-19294/thymol-exemption-from-the-requirement-of-a-tolerance.

[101] FDA (2022). 172.515 Synthetic Flavoring Substances and Adjuvants.

[102] Becerril R., Nerin C., Gomez-Lus R. (2012). Evaluation of bacterial resistance to essential oils and antibiotics after exposure to oregano and cinnamon essential oils. Foodborne Pathog. Dis..

[103] Hammer K.A., Carson C.F., Riley T.V. (2012). Effects of *Melaleuca alternifolia* (tea tree) essential oil and the major monoterpene component terpinen-4-ol on the development of single- and multistep antibiotic resistance and antimicrobial susceptibility. Antimicrob. Agents Chemother..

[104] Walsh S.E., Maillard J.Y., Russell A.D., Catrenich C.E., Charbonneau D.L., Bartolo R.G. (2003). Development of bacterial resistance to several biocides and effects on antibiotic susceptibility. J. Hosp. Infect..

[105] Lei K., Zhu Y., Chen W., Pan H.-Y., Cao Y.-X., Zhang X., Guo B.-B., Sweetman A., Lin C.-Y., Ouyang W. (2019). Spatial and seasonal variations of antibiotics in river waters in the Haihe River Catchment in China and ecotoxicological risk assessment. Environ. Int..

[106] Choi K., Kim Y., Jung J., Kim M.-H., Kim C.-S., Kim N.-H., Park J. (2008). Occurrences and ecological risks of roxithromycin, trimethoprim, and chloramphenicol in the Han River, Korea. Environ. Toxicol. Chem..

[107] Pino-Otin M.R., Langa E., Val J., Mainar A.M., Ballestero D. (2021). Impact of citronellol on river and soil environments using non-target model organisms and natural populations. J. Environ. Manag..

[108] Pino-Otin M.R., Navarro J., Val J., Roig F., Mainar A.M., Ballestero D. (2022). Spanish *Satureja montana* L. hydrolate: Ecotoxicological study in soil and water non-target organisms. Ind. Crops Prod..

[109] Botelho M.A., Bezerra Filho J.G., Correa L.L., da Cruz Fonseca S.G., Montenegro D., Gapski R., Castro Brito G.A., Heukelbach J. (2007). Effect of a novel essential oil mouthrinse without alcohol on gingivitis: A double-blinded randomized controlled trial. J. Appl. Oral Sci..

[110] Vlachojannis C., Chrubasik-Hausmann S., Hellwig E., Al-Ahmad A. (2015). A Preliminary investigation on the antimicrobial activity of listerine (r), its components, and of mixtures thereof. Phytother. Res..

[111] Escobar A., Perez M., Romanelli G., Blustein G. (2020). Thymol bioactivity: A review focusing on practical applications. Arab. J. Chem..

[112] Kavoosi G., Dadfar S.M.M., Purfard A.M. (2013). Mechanical, physical, antioxidant, and antimicrobial properties of gelatin films incorporated with thymol for potential use as nano wound dressing. J. Food Sci..

[113] Koosehgol S., Ebrahimian-Hosseinabadi M., Alizadeh M., Zamanian A. (2017). Preparation and characterization of in situ chitosan/polyethylene glycol fumarate/thymol hydrogel as an effective wound dressing. Mater. Sci. Eng. C-Mater. Biol. Appl..

[114] Jiji S., Udhayakumar S., Rose C., Muralidharan C., Kadirvelu K. (2019). Thymol enriched bacterial cellulose hydrogel as effective material for third degree burn wound repair. Int. J. Biol. Macromol..

[115] Manukumar H.M., Umesha S., Kumar H.N.N. (2017). Promising biocidal activity of thymol loaded chitosan silver nanoparticles (T-C@AgNPs) as anti-infective agents against perilous pathogens. Int. J. Biol. Macromol..

[116] Fernandez M.E., Kembro J.M., Ballesteros M.L., Caliva J.M., Marin R.H., Labaque M.C. (2019). Dynamics of thymol dietary supplementation in quail (*Coturnix japonica*): Linking bioavailability, effects on egg yolk total fatty acids and performance traits. PLoS ONE.

[117] Bacova K., Zitterl-Eglseer K., Chrastinova L., Laukova A., Madarova M., Gancarcikova S., Sopkova D., Andrejcakova Z., Placha I. (2020). Effect of thymol addition and withdrawal on some blood parameters, antioxidative defence system and fatty acid profile in rabbit muscle. Animals.

[118] Soltani M., Mohamadian S., Ebrahimzahe-Mousavi H.A., Mirzargar S., Taheri-Mirghaed A., Rouholahi S., Ghodratnama M. (2014). Shirazi thyme (*Zataria multiflora*) essential oil suppresses the expression of the epsD capsule gene in *Lactococcus garvieae*, the cause of lactococcosis in farmed fish. Aquaculture.

[119] UE On Veterinary Medicinal Products and Repealing Directive 2001/82/EC. 2019, 2019/6. https://eur-lex.europa.eu/legal-content/EN/TXT/PDF/?uri=CELEX:32019R0006.

[120] Teuber M. (2001). Veterinary use and antibiotic resistance. Curr. Opin. Microbiol..

[121] Myszka K., Schmidt M.T., Majcher M., Juzwa W., Olkowicz M., Czaczyk K. (2016). Inhibition of quorum sensing-related biofilm of *Pseudomonas fluorescens* KM121 by *Thymus vulgare* essential oil and its major bioactive compounds. Int. Biodeterior. Biodegrad..

[122] White R.L., Burgess D.S., Manduru M., Bosso J.A. (1996). Comparison of three different in vitro methods of detecting synergy: Time-kill, checkerboard, and E test. Antimicrob. Agents Chemother..

[123] Vigil A.L.M., Palou E., Parish M.E., Davidson P.M. (2005). Methods for activity assay and evaluation of results. Antimicrobials in Food.

[124] Pei R.-s., Zhou F., Ji B.-p., Xu J. (2009). Evaluation of combined antibacterial effects of eugenol, cinnamaldehyde, thymol, and carvacrol against *E. coli* with an improved method. J. Food Sci..

[125] European Comm Antimicrobial S. (2000). Terminology relating to methods for the determination of susceptibility of bacteria to antimicrobial agents. Clin. Microbiol. Infect..

[126] Hu Z.Q., Zhao W.H., Asano N., Yoda Y., Hara Y., Shimamura T. (2002). Epigallocatechin gallate synergistically enhances the activity of carbapenems against methicillin-resistant *Staphylococcus aureus*. Antimicrob. Agents Chemother..

[127] Novy P., Rondevaldova J., Kourimska L., Kokoska L. (2013). Synergistic interactions of epigallocatechin gallate and oxytetracycline against various drug resistant *Staphylococcus aureus* strains in vitro. Phytomedicine.

[128] Williamson E.M. (2001). Synergy and other interactions in phytomedicines. Phytomedicine.

[129] Peleg M., Corradini M.G., Normand M.D. (2007). The logistic (Verhulst) model for sigmoid microbial growth curves revisited. Food Res. Int..

[130] An J., Zuo G.Y., Hao X.Y., Wang G.C., Li Z.S. (2011). Antibacterial and synergy of a flavanonol rhamnoside with antibiotics against clinical isolates of methicillin-resistant *Staphylococcus aureus* (MRSA). Phytomedicine.

