# Supplementary material for: Synergistic Activity of Thymol with Commercial Antibiotics against Critical and High WHO Priority Pathogenic Bacteria
Source: Plants (Basel). 2023 May 2;12(9):1868. doi: 10.3390/plants12091868 (PMC10180827; doi:10.3390/plants12091868)
Supplement: Supplementary file 1 [file plants-12-01868-s001.zip › plants-2304134-supplementary.pdf]

Table S1. Microorganisms reference and culture conditions according to ATCC datasheets for each microorganism.

<table>
<tr><th colspan="7">Microorganism culture conditions</th></tr>
<tr><th>Microorganism</th><th>Reference</th><th>GRAM</th><th>Temperature (ºC)</th><th>Time (h)</th><th>Agar/Broth</th><th>Type of respiration</th></tr>
<tr><td>Acinetobacter baumannii</td><td>ATCC 19606</td><td>Negative</td><td>37</td><td rowspan="11">24</td><td>NU</td><td rowspan="14">Aerobic</td></tr>
<tr><td>Bacillus subtilis subsp.spizizenii</td><td>ATCC 6633</td><td rowspan="2">Positive</td><td>30</td><td rowspan="2">BHI</td></tr>
<tr><td>Enterococcus faecalis</td><td>ATCC 19433</td><td rowspan="2">37</td></tr>
<tr><td>Escherichia coli</td><td>ATCC 25922</td><td rowspan="3">Negative</td><td>TS</td></tr>
<tr><td>Klebsiella aerogenes</td><td>ATCC 13048</td><td>30</td><td rowspan="2">NU</td></tr>
<tr><td>Klebsiella pneumoniae</td><td>C6</td><td rowspan="6">37</td></tr>
<tr><td>Listeria monocytogenes</td><td>ATCC 7644</td><td>Positive</td><td rowspan="2">BHI</td></tr>
<tr><td>Pasteurella aerogenes</td><td>ATCC 27883</td><td rowspan="5">Negative</td></tr>
<tr><td>Proteus mirabilis</td><td>ATCC 35659</td><td rowspan="2">TS</td></tr>
<tr><td>Pseudomonas aeruginosa</td><td>ATCC 27853</td></tr>
<tr><td>Salmonella typhimurium</td><td>ATCC 13311</td><td rowspan="2">NU</td></tr>
<tr><td>Serratia marcescens subsp.marcescens</td><td>ATCC 13880</td><td>26</td><td>24-48</td></tr>
<tr><td>Staphylococcus aureus</td><td>ATCC 9144</td><td rowspan="2">Positive</td><td rowspan="2">37</td><td rowspan="2">24</td><td>TS</td></tr>
<tr><td>Streptococcus agalactiae</td><td>ATCC 12386</td><td>BHI</td></tr>
<tr><td colspan="7">TS -Trypticase Soy Agar/Broth, NU - Nutrient agar or nutrient broth, BHI - Brain Heart Infusion Agar/Broth.</td></tr>
</table>

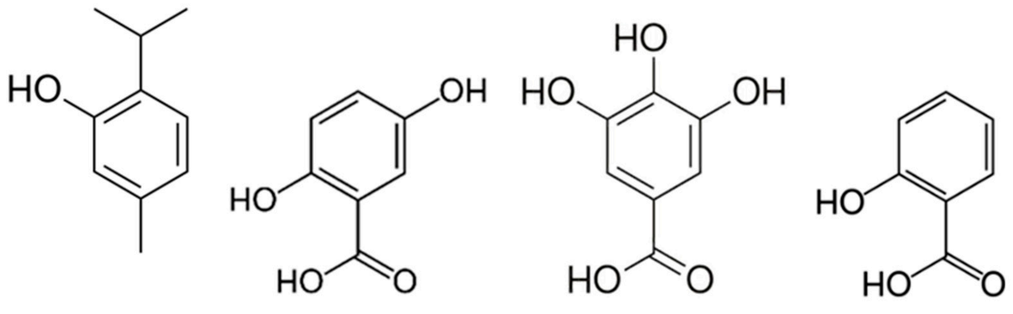

Thymol Gentisic acid Gallic acid Salicylic acid

Figure S1. Chemical structures of the tested natural products.
